# Multi-trajectories of serum uric acid/high density lipoprotein cholesterol ratio and fasting plasma glucose in chronic kidney disease

**DOI:** 10.1080/07853890.2026.2689825

**Published:** 2026-06-18

**Authors:** Mingxuan Xie, Zhenghong Luo, Huixian Xiong, Juan Jiang, Yuping Liu, Shujin Guo, Xiaopeng Yao, Zhengwei Wan, Junlin Zhang, Ping Shuai

**Affiliations:** a Department of Health Management & Physical Examination, Sichuan Provincial People’s Hospital, University of Electronic Science and Technology of China, Chengdu, China; b School of Public Health, Southwest Medical University, Luzhou, China; c School of Medical Information and Engineering, Southwest Medical University, Luzhou, China; d School of Medicine, University of Electronic Science and Technology of China, Chengdu, China

**Keywords:** Chronic kidney disease, serum uric acid/high density lipoprotein cholesterol ratio, fasting plasma glucose, Group-based multi trajectory models

## Abstract

**Background:**

This study aimed to investigate the associations of longitudinal trajectories of the serum uric acid to high-density lipoprotein cholesterol ratio (UHR) and fasting plasma glucose (FPG) with chronic kidney disease (CKD) risk.

**Methods:**

In this retrospective cohort study, we included 7,541 adults who underwent at least three annual health examinations. Cox proportional hazards models were used to evaluate associations of UHR and FPG (in quartiles) with incident CKD. Group-based multi-trajectory modeling (GBMTM) was subsequently applied to identify distinct joint trajectories of UHR and FPG.

**Results:**

During follow-up, 558 participants developed CKD (incidence 7.40%). In fully adjusted Cox models, higher UHR quartiles remained independently associated with increased CKD risk. The association for FPG was attenuated after full adjustment.GBMTM identified four joint trajectory groups: Group 1(low UHR–low FPG), Group 2(moderate UHR–moderate FPG), Group 3(moderate–high UHR with fluctuating moderate FPG), and Group 4(high UHR with fluctuating moderate FPG). Compared to Group 1, the adjusted hazard ratios (95% CI) for CKD were 1.34 (1.05 ∼ 1.70) for Group 2, 2.31 (1.73 ∼ 3.09) for Group 3, and 4.21 (2.88 ∼ 6.15) for Group 4. Subgroup analyses generally supported these findings.

**Conclusions:**

While elevated UHR is independently associated with the risk of developing CKD, the association for FPG was attenuated after full adjustment. The joint trajectory analysis highlights that their dynamic co-evolution is significantly associated with CKD risk, identifying a high-risk phenotype that may inform early risk stratification.

## Graphical Abstract

**Figure:**
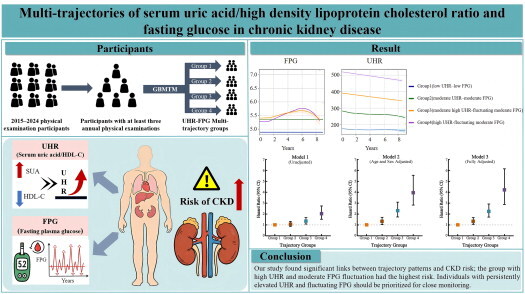

## Introduction

Chronic kidney disease (CKD), characterized by its high prevalence, elevated mortality, low awareness, and substantial economic burden, has emerged as a pressing global public health challenge [1]. Epidemiological data from the Kidney Disease Improving Global Outcomes(KDIGO) consortium revealed an increasing trend in the disease burden of CKD over the past three decades. Between 1990 and 2017, the global incidence of CKD increased by 29.3%, accompanied by a more significant 41.5% increase in CKD-associated mortality [2]. The increasing prevalence of CKD, coupled with the concurrent and sharp increase in mortality rates and healthcare expenditures, has placed a substantial burden on healthcare systems worldwide [3]. However, a major practical difficulty in managing CKD lies in its insidious onset and low public awareness. Traditional diagnostic markers, such as a decline in estimated glomerular filtration rate (eGFR) or the presence of microalbuminuria, often indicate that irreversible renal structural damage has already occurred [4]. Consequently, identifying accessible, cost-effective early warning biomarkers to facilitate the early identification of CKD before overt renal impairment manifests is a critical clinical gap that needs to be addressed.

In the early pathogenesis of CKD, metabolic disorders play a central role, and abnormal changes in biomarkers are often important signals of metabolic imbalance. A novel integrative biomarker, Serum uric acid(SUA)/high density lipoprotein cholesterol(HDL-C) ratio(UHR), comprehensively reflects the imbalance in the oxidative-inflammatory-metabolic network. It has shown remarkable advantages, particularly in the early warning and risk assessment of metabolic diseases [5]. Previous studies have confirmed that elevated UHR increase the risks of metabolic syndrome [6], type 2 diabetes [7], and nonalcoholic fatty liver disease [8]. As a core biomarker of dysglycemia, long-term fluctuations in fasting plasma glucose (FPG) not only drive the progression of diabetic nephropathy but also mediate renal parenchymal damage in non-diabetic patients through pathways involving advanced glycosylation end-products (AGEs) accumulation and mitochondrial oxidative stress [9].

Remarkably, UHR and FPG may establish a bidirectional pathophysiological interplay: clinical studies suggest that hyperuricemia may exacerbate blood glucose fluctuations by interfering with insulin signaling pathways, whereas a hyperglycemic environment not only promotes the production of uric acid but also inhibits its excretion, thereby establishing a vicious cycle of metabolic imbalance [10]. Given that FPG, SUA, and HDL-C are routine, easily accessible tests in most health examination centers, integrating these markers could provide an economical and highly translational tool for early CKD risk assessment.

Despite this clinical potential, a major limitation of the current literature is its heavy reliance on cross-sectional analyses or single-time-point measurements of individual biomarkers. While recent epidemiological studies have established an association between elevated baseline or cumulative UHR and increased CKD risk, these evaluations inherently fail to capture the dynamic, co-evolving nature of metabolic imbalances over time. Furthermore, investigating UHR or FPG in isolation overlooks their synergistic pathophysiological effects. To address this gap, this 10-year retrospective cohort study utilized Group-Based Multi-Trajectory Modeling (GBMTM) to investigate the joint longitudinal trajectories of UHR and FPG. The incremental value of this joint trajectory approach lies in its ability to simultaneously capture the longitudinal co-evolution of two bidirectionally linked biomarkers. By shifting the research paradigm from static, single-marker assessment to dynamic joint monitoring, this study aims to identify distinct, high-risk clinical phenotypes that would remain completely obscured in traditional analyses, ultimately providing a reference basis for the early screening and prevention of CKD.

## Methods

### Subjects of the study

We conducted a retrospective cohort study of adults from the annual health examination database of the Health Management Center at Sichuan Provincial People’s Hospital (January 1,2015 to December 31, 2024). The study population comprised participants with at least three health surveys and complete data on eGFR, UACR, FPG, HDL-C, and SUA. We excluded individuals based on the following operationalized criteria: (1) A history of liver cirrhosis, severe infections, malignant tumors, mental disorders, receipt of hemodialysis or peritoneal dialysis, prior kidney transplantation, or previous nephrectomy (ascertained *via* standardized self-reported medical history questionnaires); and (2) Baseline renal function abnormalities, defined operationally as having an eGFR < 60 mL/min/1.73 m^2^ or a urine albumin-to-creatinine ratio (UACR) ≥ 30 mg/g at their initial health examination. After applying these criteria and removing records with missing key variables, 7,541 individuals were retained for the final analysis. The participant selection process is detailed in Figure S1 in the Supplementary Material. The study protocol was approved by the Ethics Committee of Sichuan Provincial People’s Hospital (Approval No. 2025 − 387).

Study timeline: Health examination data were collected for the period from January 1, 2015, to December 31, 2024. Data extraction and screening were performed in March 2025. Statistical analysis was conducted from April to August 2025.

### Questionnaires

We collected basic demographic information, lifestyle factors, and disease history using a standardized hospital-based questionnaire. Lifestyle factors included alcohol consumption status, which was defined based on self-reported drinking habits. Participants who reported consuming an average of at least one alcoholic drink per week for a minimum of six months were classified as drinkers; all others were considered nondrinkers [11].

### Physical examination

The diastolic blood pressure (DBP) and systolic blood pressure (SBP) of the study subjects in the resting seated position were measured by healthcare personnel who had undergone standardized training *via* the Omron HBP-9020 fully automatic electronic sphygmomanometer. Measurements were conducted at 2-minute intervals, and the average of three readings was determined. Height and weight data were collected using the SK-14B0121 electronic height-weight measuring instrument. The body mass index (BMI) was then calculated *via* the following formula: BMI = weight/height^2^ (kg/m^2^) for further evaluation.

### Biochemical examination

Blood biochemical measurements including: FPG(mmol/L), total cholesterol (TC, mmol/L), triglyceride (TG, mmol/L), HDL-C(mmol/L), low-density lipoprotein cholesterol (LDL-C, mmol/L), SUA (μmol/L), serum creatinine (SCr, μmol/L)and blood urea nitrogen (BUN, mmol/L), were performed via the Roche c702 fully automated biochemical analyzer.

Urine samples were collected from all participants and analyzed using an automated biochemical analyzer to quantify urine albumin and creatinine concentrations. The UACR was calculated as urine albumin (mg/L) divided by urine creatinine (g/L). The eGFR was calculated using the 2021 Chronic Kidney Disease Epidemiology Collaboration (CKD-EPI) creatinine equation [12]^.^ The UHR was calculated using the exact formula established in previous studies: UHR = SUA (μmol/L)/HDL-C (mmol/L) [13].

### Definitions

(1) The primary outcome was operationally defined incident CKD. Specifically, for renal abnormalities (eGFR <60 mL/min/1.73 m^2^ or UACR ≥30 mg/g) detected during intermediate follow-ups, we utilized data from the subsequent examination to verify persistence; participants whose abnormalities persisted across consecutive visits were classified as having incident CKD. For cases where the first abnormality occurred at the final follow-up visit, incident CKD was operationally defined based on the single-time-point measurement [14,15]. Regarding event dating for survival analyses, the date of the CKD event was assigned to the date of the first abnormal visit for cases confirmed during intermediate follow-ups, and to the date of the final visit for single-measurement cases.(2) According to the Chinese Guidelines for the Prevention and Treatment of Hypertension, hypertension is defined as follows: SBP ≥ 140 mmHg and/or DBP ≥ 90 mmHg (1 mmHg = 0.133 kPa), and/or individuals with a previous history of hypertension who are currently taking antihypertensive medications [16].(3) According to the Chinese Guidelines for the Prevention and Treatment of Type 2 Diabetes, diabetes mellitus is defined as follows: FPG ≥ 7.0 mmol/L, glycated hemoglobin (HbA1c)≥6.5%, or individuals with a previous diagnosis of diabetes mellitus [17]. (4) According to the Joint Committee on the Chinese Guidelines for Lipid Management, dyslipidemia is defined as follows: TC concentration ≥ 5.18 mmol/L, TG concentration ≥ 1.70 mmol/L, LDL-C concentration ≥ 3.37 mmol/L, HDL-C concentration < 1.04 mmol/L, and/or the current use of lipid-regulating drugs [18]. (5) According to the Chinese Guidelines for the Diagnosis and Treatment of Hyperuricemia and Gout, hyperuricemia (HUA) is defined as follows: SUA level ≥ 420 μmol/L in men or ≥ 360 μmol/L in women [19].

### Statistical analysis

We performed all statistical analyses using SAS(version 9.4) and R(version 4.4.2). We summarized baseline characteristics according to data type and distribution. For continuous variables with a normal distribution, we present means with standard deviations (SD) and used independent samples t-tests for comparisons between two groups and one-way analysis of variance (ANOVA) for comparisons across multiple groups. For continuous variables with a non-normal distribution, we present medians with interquartile ranges (IQR) and used the Wilcoxon rank-sum test for two-group comparisons and the Kruskal-Wallis test for multi-group comparisons. We expressed categorical variables as frequencies (%) and compared them using the chi-square (χ^2^) test.

We calculated follow-up time from the date of the baseline examination until the assigned date of incident CKD (as defined above), or the date of the last available health examination for those who did not develop CKD, whichever came first. To assess the associations of the UHR and FPG with incident CKD, we fitted Cox proportional hazards regression models. The proportional hazards assumption for the primary exposures (trajectory groups, UHR quartiles, and FPG quartiles) was rigorously validated using Schoenfeld residuals. All individual P-values exceeded 0.05 (trajectory groups: *p* = 0.55; UHR quartiles: *p* = 0.46; FPG quartiles: *p* = 0.23), confirming that the proportional hazards assumption was fully satisfied for the key variables of interest. We explored potential nonlinear dose-response relationships by incorporating restricted cubic splines (RCS) into the fully adjusted Cox models. For the RCS analysis, we placed three knots at the specified percentiles (10th, 50th, and 90th) of the biomarker distributions. We tested both the P for overall association and the P for non-linearity. We conducted prespecified subgroup analyses stratified by sex, age, alcohol consumption status, hypertension, and BMI.

We identified trajectory changes in UHR combined with FPG by the Group-Based Multi-Trajectory Modeling (GBMTM), and trajectory modeling was achieved by the PROC TRAJ process developed by Nagin [20] and his colleagues based on SAS. Adhering to the goals of model applicability and parsimony, we constructed the cluster multi-trajectory model by combining specialized interpretability through model fit effectiveness metrics. Considering that both UHR and FPG are continuous variables, the Censored Normal (CNORM) distribution was utilized for modeling. The model fit effectiveness metrics include Average posterior probability (Avep%), Proportions per class, Odds of correct classification (OCC) and Bayesian Information Criterion (BIC). We fitted linear, squared, and cubed trajectory models for groups 1–5, respectively, and chose the optimal number of trajectory groups by comparing the fit metrics of different models and the professional interpretability of the model trajectory morphology: i) BIC was as close to 0 as possible; ii) Avep > 0.7 in each group; iii) OCC > 5.0 in each group; and iv) each group contained at least 5% of the participants [21,22].

The trajectory-building window utilized all available biomarker measurements across the entire 10-year follow-up period to maximize the characterization of long-term metabolic patterns; hence, post-event measurements were not excluded from the initial PROC TRAJ modeling. The requirement for participants to have at least three annual health examinations was strictly applied because defining a longitudinal trajectory mathematically necessitates a minimum of three distinct data points. To robustly accommodate the varying numbers of annual measurements (ranging from 3 to 10 waves per participant) and irregular follow-up intervals, the actual follow-up time (exact years from baseline) was utilized as the continuous time-scale variable in both trajectory modeling and Cox regression. Missing data were handled natively *via* maximum likelihood estimation under the Missing at Random (MAR) assumption. Furthermore, because this overlapping temporal design may introduce a potential bias of reverse causality, we conducted stepwise sensitivity analyses. We reevaluated the fully adjusted Cox models by sequentially excluding early incident CKD cases diagnosed at the second follow-up visit (as they lacked the minimum three pre-disease data points to robustly define a trajectory), and subsequently excluding cases diagnosed up to the third follow-up visit. *p* < 0.05 was considered statistically significant.

## Results

### Baseline characteristics of the study population

A total of 7,541 participants (mean age 49.91 ± 14.29 years; 58.04% male) were included in this study. During follow-up, 558 incident CKD cases were identified, yielding an incidence rate of 7.40%. Participants were stratified into two groups based on CKD occurrence for univariate analysis. Compared to the non-CKD group, participants who developed CKD were older and had significantly higher levels of SBP, BMI, SUA, UACR, UHR, and FPG, as well as a higher prevalence of diabetes, hyperuricemia and hypertension (all *p* < 0.05). Conversely, the CKD group exhibited significantly lower levels of TC, LDL-C, HDL-C, and eGFR (all *p* < 0.05). No statistically significant differences were observed in DBP, TG, sex distribution, or the proportion of alcohol drinkers between the two groups as presented in Table 1.

**Table 1.** Baseline characteristics of the study population according to incident CKD status.

Characteristics	Total (*n* = 7541)	Normal group (*n* = 6983)	CKD group (*n* = 558)	Statistic	*P*
Age, years	49.91 ± 14.29	49.18 ± 13.74	59.13 ± 17.42	t=-13.17	**<0.001**
SBP, mmHg	120.35 ± 16.10	119.70 ± 15.69	128.61 ± 18.69	t=-10.94	**<0.001**
DBP, mmHg	72.22 ± 10.61	72.17 ± 10.56	72.90 ± 11.32	t=-1.56	0.119
BMI, kg/m^2^	23.66 ± 3.09	23.63 ± 3.09	24.03 ± 2.97	t=-2.93	**0.003**
TC, mmol/L	4.78 ± 0.91	4.79 ± 0.90	4.63 ± 0.97	t = 3.93	**<0.001**
TG, mmol/L	1.60 ± 1.23	1.59 ± 1.21	1.69 ± 1.44	t=-1.92	0.055
LDL-C, mmol/L	2.78 ± 0.80	2.79 ± 0.79	2.70 ± 0.87	t = 2.40	**0.017**
HDL-C, mmol/L	1.34 ± 0.32	1.34 ± 0.32	1.25 ± 0.31	t = 6.28	**<0.001**
SUA, μmol/L	342.06 ± 85.42	340.26 ± 84.86	364.59 ± 89.24	t=-6.49	**<0.001**
UACR, mg/g	7.97 ± 5.56	7.68 ± 5.27	11.62 ± 7.48	t=-12.22	**<0.001**
eGFR, ml/min	101.70 ± 15.83	102.60 ± 14.91	90.44 ± 21.57	t = 13.07	**<0.001**
BUN, mmol/L	5.17 ± 1.25	5.15 ± 1.24	5.43 ± 1.37	t=-4.70	**<0.001**
SCr, μmol/L	68.50 ± 14.96	68.01 ± 14.50	74.53 ± 18.84	t=-7.98	**<0.001**
UHR	277.96 ± 115.90	274.89 ± 114.11	316.32 ± 130.44	t=-7.28	**<0.001**
FPG, mmol/L	5.12 ± 1.08	5.09 ± 1.03	5.49 ± 1.50	t=-6.14	**<0.001**
Male, n (%)	4,377 (58.04)	4,041 (57.87)	336 (60.22)	χ²=1.17	0.280
Hypertension, n (%)	1,556 (20.63)	1,317 (18.86)	239 (42.83)	χ²=181.31	**<0.001**
Type 2 diabetes, n (%)	597 (7.92)	500 (7.16)	97 (17.38)	χ²=74.08	**<0.001**
Hyperuricemia, n (%)	1,610 (21.35)	1,435 (20.55)	175 (31.36)	χ²=35.97	**<0.001**
Dyslipidemia, n (%)	4182 (55.46)	3846 (55.08)	336 (60.22)	χ²=5.52	**0.019**
Alcohol drinker, n (%)	1,081 (14.33)	987 (14.13)	94 (16.85)	χ²=3.09	0.079

Note: Bold values indicate statistical significance (*P* < 0.05).

### Results of cox regression analysis of UHR and FPG with CKD

**Figure 1.**
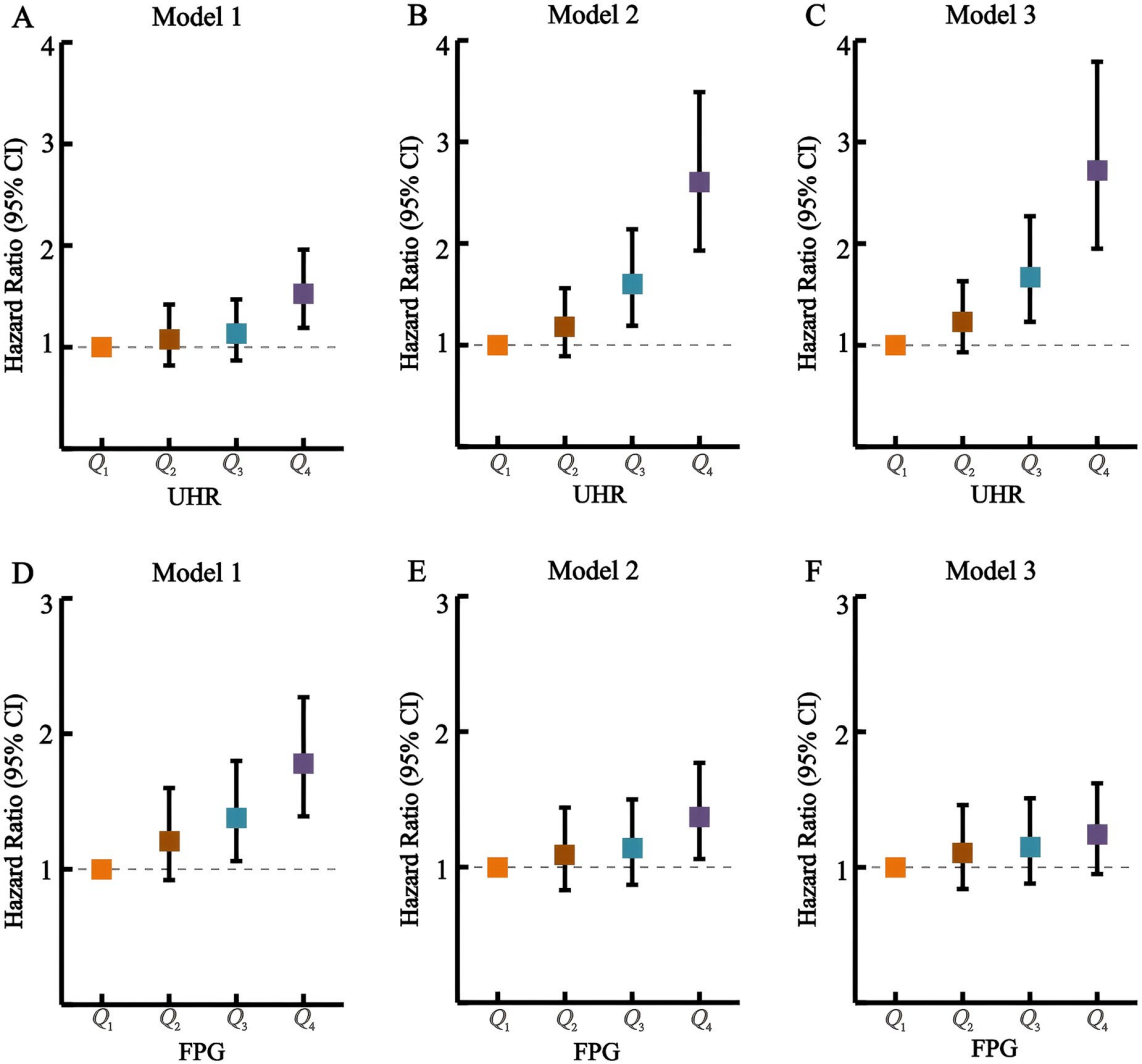
Association of UHR and FPG quartiles with CKD risk: (A–C) Display hazard ratios (HRs) and 95% confidence intervals (CIs) for CKD risk across UHR quartiles (*Q*_1_ to *Q*_4_); (D–F) Display HRs and 95% CIs for CKD risk across FPG quartiles (*Q*_1_ to *Q*_4_).

The associations between UHR and FPG quartiles (*Q*_1_-*Q*_4_, with *Q*_1_ as reference) and CKD incidence was evaluated using Cox proportional hazards regression. For UHR: Unadjusted analysis revealed a significantly higher risk in *Q*_4_ vs *Q*_1_ (*HR* = 1.53, 95% *CI*: 1.19 ∼ 1.96, *p* < 0.001). Adjustment for sex and age demonstrated significantly elevated risks in both *Q*_3_ (*HR* = 1.60, 95% *CI*: 1.19 ∼ 2.14, *p* < 0.05) and *Q*_4_ (*HR* = 2.60, 95% *CI*: 1.93 ∼ 3.49, *p* < 0.001). Full adjustment for alcohol consumption, hypertension, SBP, LDL-C, TC, BMI, BUN/Cr Ratio, and FPG maintained these associations. For FPG: Unadjusted analysis showed significantly elevated risks in *Q*_3_ and *Q*_4_ (both *p* < 0.05). Adjustment for sex and age retained significance in *Q*_4_ (*HR* = 1.37, 95% *CI*: 1.06 ∼ 1.77, *p* = 0.017). Full adjustment for alcohol consumption, hypertension, SBP, LDL-C, TC, BMI, BUN/Cr Ratio, and UHR further attenuated the associations, with no quartile showing a significant difference from the reference (Table 2 and Figure 1). Additionally, RCS analysis was employed to evaluate the shape of the dose-response trends (Figure S2). For UHR, there was a significant overall association with incident CKD *(*P for overall association < 0.001), and the trend was linear (*P* for non-linearity > 0.05). For FPG, while the curve exhibited a linear shape (*P* for non-linearity > 0.05), its independent association was attenuated after full adjustment, which is consistent with the findings in the fully adjusted quartile models.

**Table 2.** Results of cox regression analysis of UHR and FPG quartile groupings with CKD.

Variables and Grouping	Model 1		Model 2		Model 3	
	*HR* (95% *CI)*	*P*	*HR* (95% *CI)*	*P*	*HR* (95% *CI)*	*P*
UHR						
*Q*_1_	1.00 (Reference)		1.00 (Reference)		1.00 (Reference)	
*Q*_2_	1.08 (0.82–1.42)	0.583	1.18 (0.89–1.56)	0.240	1.23 (0.93–1.63)	0.179
*Q*_3_	1.13 (0.87–1.47)	0.365	1.60 (1.19–2.14)	**0.002**	1.67 (1.23–2.27)	**<0.001**
*Q*_4_	1.53 (1.19–1.96)	**<0.001**	2.60 (1.93–3.49)	**<0.001**	2.72 (1.95–3.79)	**<0.001**
FPG						
*Q*_1_	1.00 (Reference)		1.00 (Reference)		1.00 (Reference)	
*Q*_2_	1.21 (0.92–1.60)	0.161	1.09 (0.83–1.44)	0.527	1.11 (0.84–1.46)	0.468
*Q*_3_	1.38 (1.06–1.80)	**0.017**	1.14 (0.87–1.50)	0.335	1.15 (0.88–1.51)	0.313
*Q*_4_	1.78 (1.39–2.27)	**<0.001**	1.37 (1.06–1.77)	**0.017**	1.24 (0.95–1.62)	0.111

Note: Model 1 unadjusted; Model 2 adjusted for age, sex; Model 3 adjusted for further corrected for alcohol consumption, hypertension, SBP, LDL-C, TC, BMI, and BUN/Cr Ratio on the basis of Model 2, and adjusted for FPG when analyzing UHR, and UHR when analyzing FPG. Bold values indicate statistical significance (*P* < 0.05).

### Optimal model selection process for UHR-FPG multi-trajectory groups

**Figure 2.**
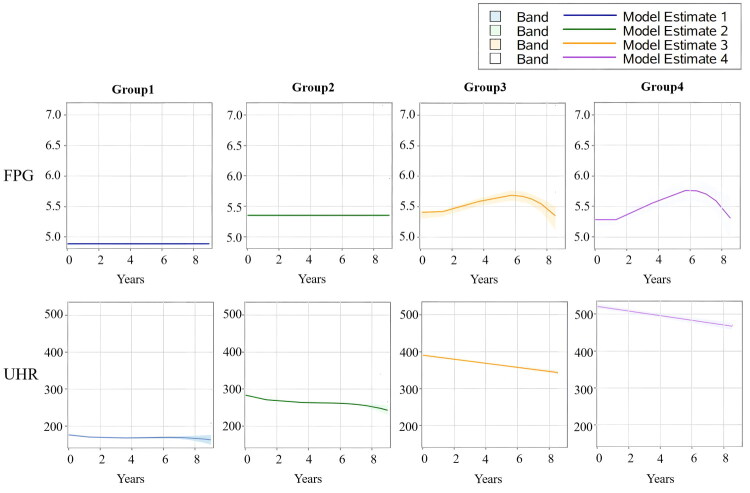
UHR-FPG multi-trajectory groups.

We adopt the GBMTM model to identify multi-trajectories changes in the joint UHR and FPG, based on the PROC TRAJ process developed by SAS for trajectory modeling. For optimal model selection, we fitted models with 1 to 5 potential trajectory groups (testing different functional forms) and screened the best model based on the criteria predefined in the Methods section. The optimal model was confirmed to include 4 distinct joint trajectory groups, named by their characteristic trends: Group 1 (low UHR–low FPG), Group 2 (moderate UHR–moderate FPG), Group 3 (moderate–high UHR with fluctuating moderate FPG), and Group 4 (high UHR with fluctuating moderate FPG). The trajectory trends of each group are shown in Figure 2. Detailed tables of model fit evaluation metrics are provided in the supplementary Tables S1–S3.

**Table 3.** Cox regression analysis of UHR-FPG multi-trajectory groups.

Trajectory Groups	Model 1 *HR* (95% *CI*)	*P*	Model 2 *HR* (95% *CI*)	*P*	Model 3 *HR* (95% *CI*)	*P*
Group 1	1.00 (Reference)		1.00 (Reference)		1.00 (Reference)	
Group 2	1.07 (0.87–1.32)	0.528	1.33 (1.06–1.68)	**0.014**	1.34 (1.05–1.70)	**0.019**
Group 3	1.35 (1.08–1.68)	**0.008**	2.24 (1.72–2.92)	**<0.001**	2.31 (1.73–3.09)	**<0.001**
Group 4	2.04 (1.52–2.74)	**<0.001**	3.96 (2.82–5.56)	**<0.001**	4.21 (2.88–6.15)	**<0.001**

Note: Model 1 unadjusted; Model 2 was adjusted for age and sex; Model 3 was further adjusted for alcohol, hypertension, SBP, LDL-C, TC, BUN/Cr Ratio and BMI on the basis of Model 2. Unlike the quartile analyses, UHR and FPG were not included as covariates in this model because they define the trajectory groups. Bold values indicate statistical significance (*P* < 0.05).

### Baseline characteristics and cumulative CKD incidence in UHR–FPG trajectory groups

**Figure 3.**
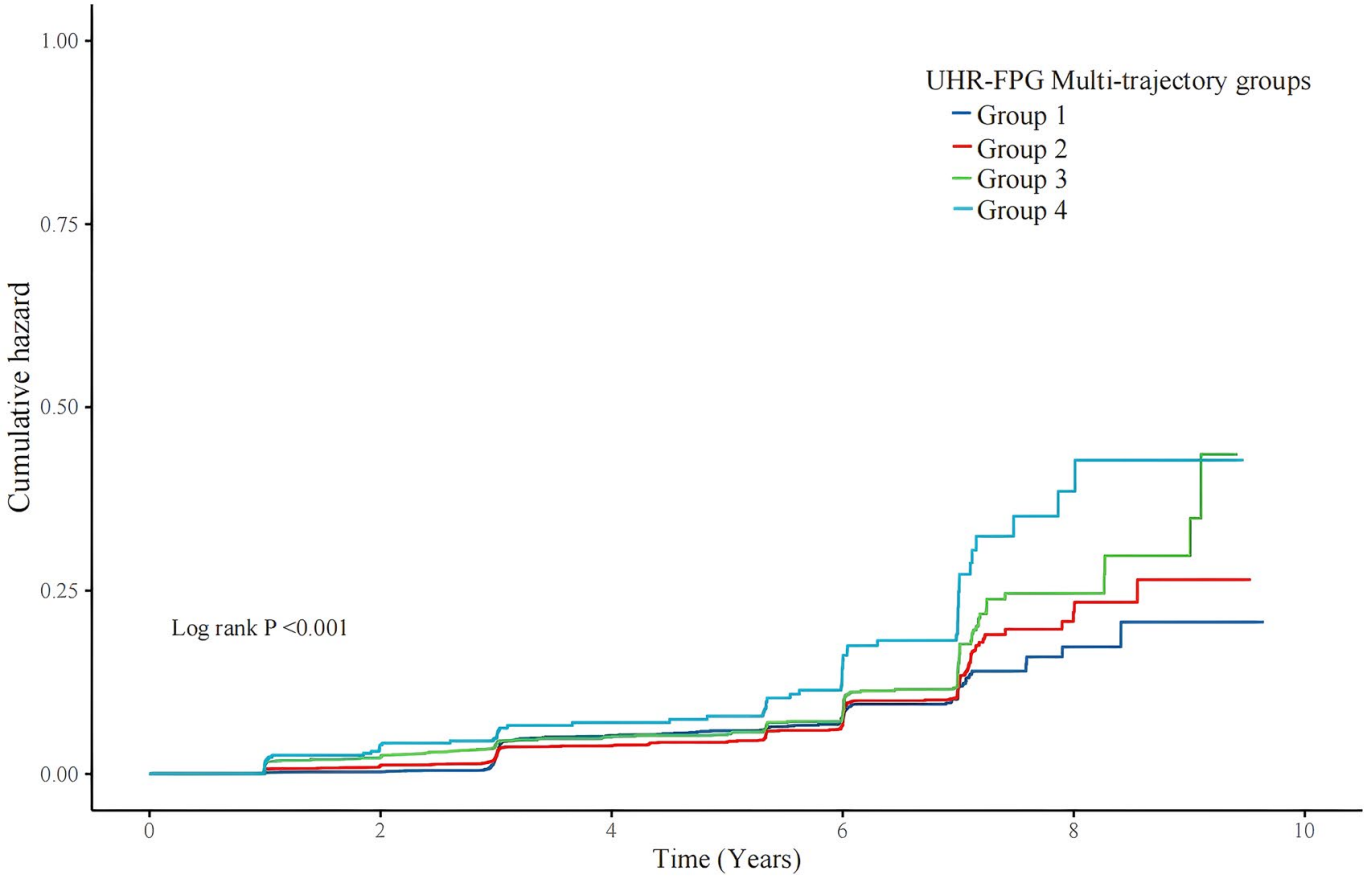
Kaplan-Meier curves of cumulative risk across four UHR-FPG multi-trajectory groups.

Participants were stratified into four distinct multi-trajectory groups based on their UHR and FPG patterns over the 10-year follow-up: Group 1, low UHR–low FPG (*n* = 3,072, 40.7%); Group 2, moderate UHR–moderate FPG (*n* = 2,473, 32.8%); Group 3, moderately-high UHR with fluctuating moderate FPG (*n* = 1,598, 21.2%); and Group 4, high UHR with fluctuating moderate FPG (*n* = 398, 5.3%). Groups differed significantly in age, cardiometabolic risk factors, and renal parameters (all *p* < 0.001), with Group 4 exhibiting the highest BMI, SUA, UHR, and prevalence of hyperuricemia (Table S4). Correspondingly, the cumulative risk over the 10-year follow-up differed significantly among the four trajectory groups (log-rank test, *p* < 0.001; Figure 3). Group 1 maintained the lowest risk throughout, while Group 4 exhibited the steepest increase, with a risk that was substantially higher than all other groups in later follow-up. The risks for Groups 2 and 3 were intermediate.

### Multifactorial analysis of the risk of developing CKD in the UHR-FPG multi-trajectory groups

**Figure 4.**
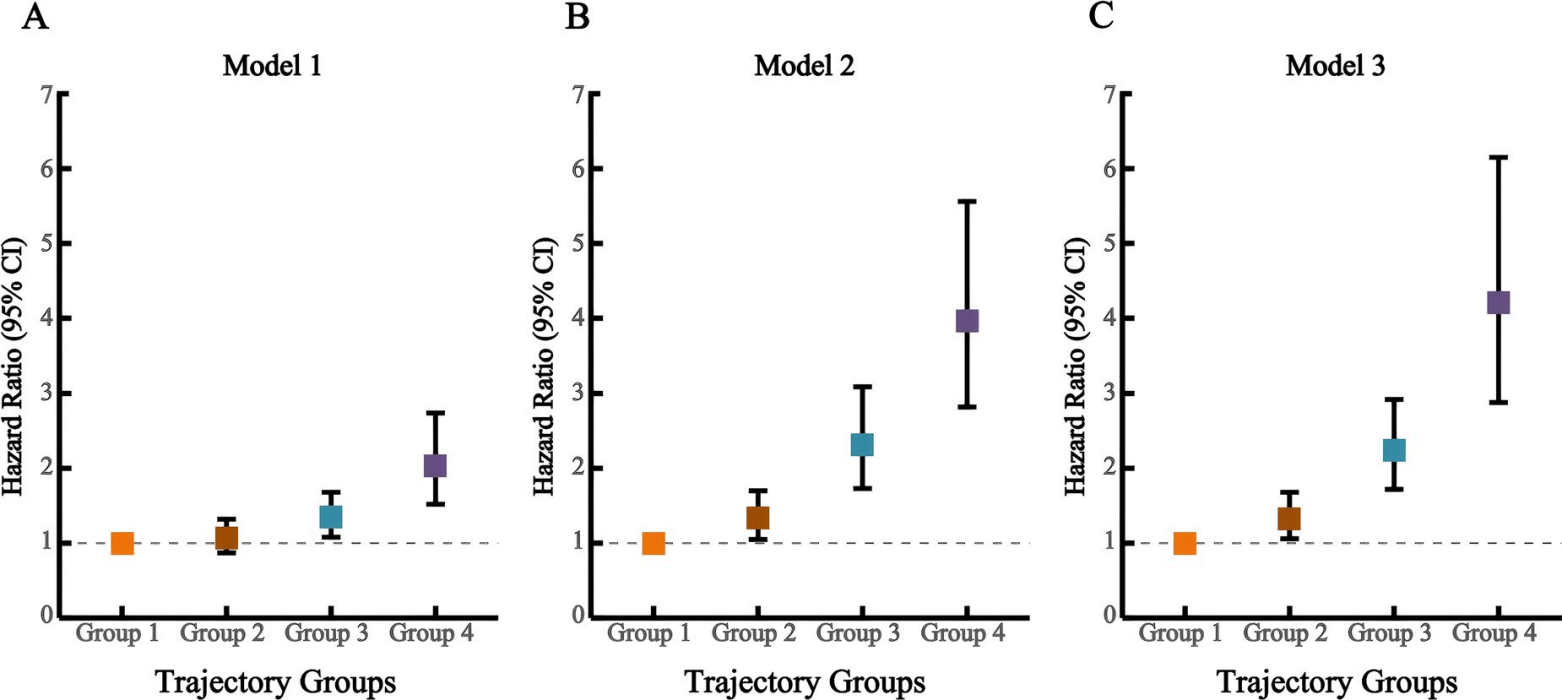
Association of trajectory groups with incident CKD: (A–C) Present hazard ratios (HRs) and 95% confidence intervals (CIs) for CKD risk across trajectory groups (Group 1 to Group 4) in three Cox models.

Using Group 1 as the reference, Cox regression analysis revealed that, without adjustment for confounders, Groups 3 (*HR* = 1.35, 95% *CI*: 1.08–1.68) and 4 (*HR* = 2.04, 95% *CI*: 1.52–2.74) had a significantly higher risk of incident CKD compared with Group 1 (all *p* < 0.05). After adjusting for age and sex, the risk was significantly increased in Group 2 (*HR* = 1.31, 95% *CI*: 1.06–1.68, *p* = 0.014), and further increased in Group 3 (*HR* = 2.24) and Group 4 (*HR* = 3.96) (both *p* < 0.001). After further adjustment for alcohol consumption, hypertension, SBP, LDL-C, TC, BMI and BUN/Cr Ratio, this relationship persisted, with Group 4 having a 4.21 times higher risk of CKD onset than Group 1 (*HR* = 4.21, 95% *CI*: 2.88–6.15, *p* < 0.001), as shown in Table 3 and Figure 4. Furthermore, to mitigate potential bias from reverse causality, we performed stepwise sensitivity analyses. Reassuringly, the associations between the UHR-FPG trajectory groups and incident CKD risk remained highly robust after sequentially excluding early incident cases diagnosed at the second follow-up visit, and up to the third follow-up visit. In both restricted models, participants in Group 4 consistently exhibited the highest risk of incident CKD compared to Group 1 (all *p* < 0.001) (Supplementary Table S5).

### Subgroup analysis of UHR-FPG multi-trajectory groups and risk of CKD onset

**Figure 5.**
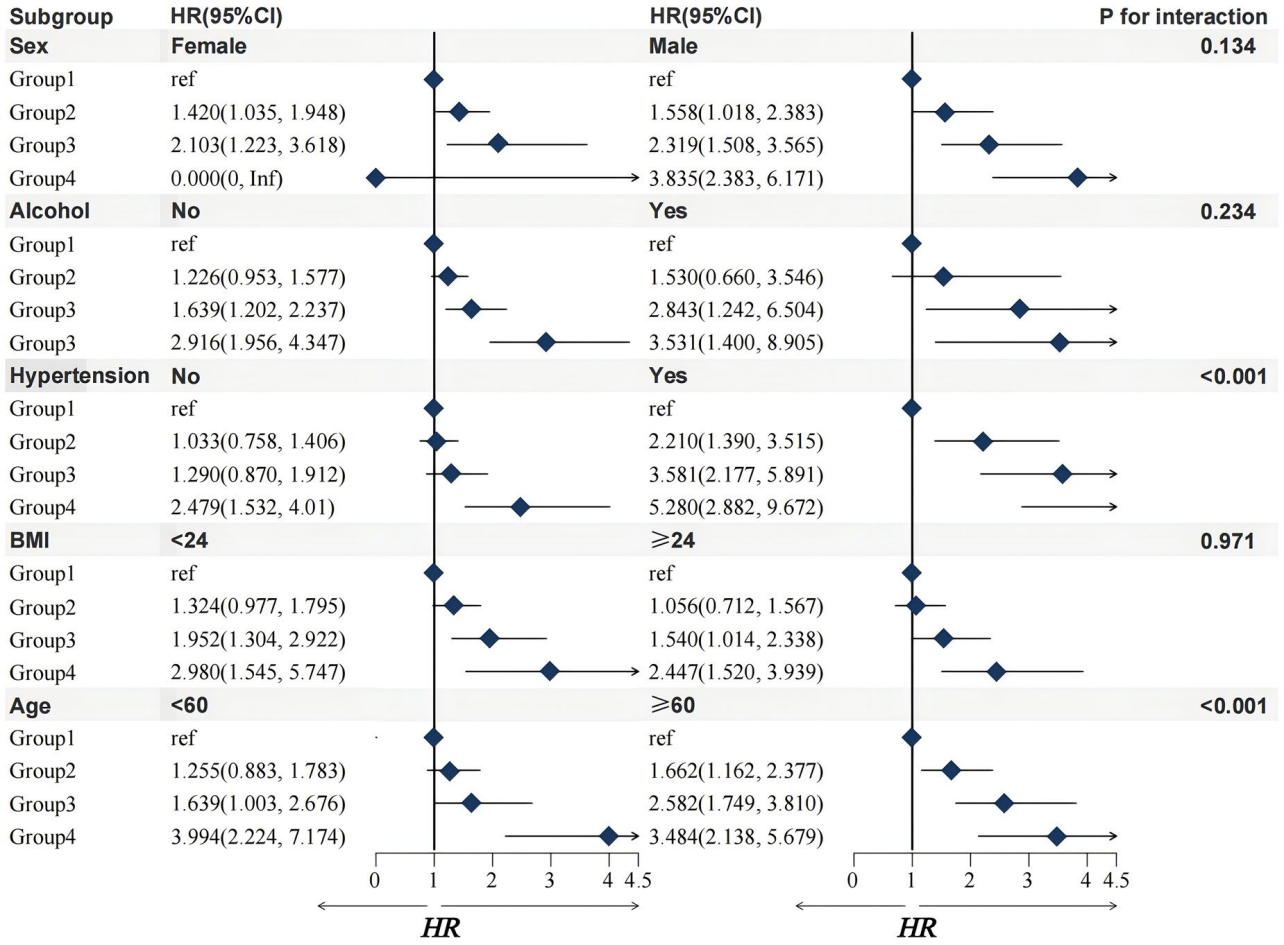
Subgroup analysis of UHR-FPG Multi-trajectory groups and risk of CKD development: The covariates adjusted in the models included sex, age, BMI, SBP, TC, LDL-C, BUN/Cr Ratio, hypertension, and alcohol consumption. The stratification factor itself was excluded from the adjustment model in its corresponding subgroup analysis.

Subgroup analysis of different genders, ages, BMI, hypertension, and alcohol consumption showed that UHR-FPG trajectory grouping was correlated with the risk of CKD development in certain subgroups. Among men, hypertension, and age ≥60 years, the risk of CKD onset was significantly higher in Group 2, Group 3, and Group 4, using Group 1 as the reference group (all *p* < 0.05). In contrast, among those aged <60 years, drinkers, nondrinkers, BMI ≥ 24 (kg/m^2^) and BMI < 24 (kg/m^2^), the risk of developing CKD was increased in Group 3 and Group 4 compared to Group 1, with a statistically significant difference (all *p* < 0.05). Of all subgroups, Group 4 had the highest risk of morbidity, suggesting that the joint trajectory pattern of high UHR with fluctuating FPG was strongly associated with CKD risk. In contrast, the *HR* for the female subgroup in Group 4 could not be reliably estimated due to an exceptionally small sample size (*n* = 8, representing only 2% of the group) and the absence of incident CKD events. Consequently, the risk estimation for this specific sex subgroup is statistically unreliable and should be interpreted with caution. Subgroup analyses revealed significant interactions between hypertension and age subgroups and trajectory groups (both *p* < 0.05), as shown in Figure 5.

## Discussion

In this 10-year retrospective cohort study, we employed GBMTM to characterize the longitudinal co-trajectories of the UHR and FPG and to evaluate their associations with the risk of incident CKD.

Single-marker Cox regression analyses revealed initial associations between both UHR and FPG and the risk of incident CKD. After adjusting for age and sex, with UHR *Q*_1_ as the reference group, individuals in UHR *Q*_3_ and *Q*_4_ exhibited significantly elevated CKD risk (both *p* < 0.05). This association remained statistically significant following further adjustment for additional covariates: the risk of incident CKD in UHR *Q*_4_ was 2.72 times that of *Q*_1_ (*HR* = 2.72, 95% *CI*: 1.95–3.79). Our result is consistent with previous studies that have identified UHR as a pertinent risk marker for CKD. For example, a prior cohort study reported a 28% increase in CKD risk per standard deviation increase in cumulative UHR [23], and a cross-sectional analysis in a Chinese middle-aged and elderly population found that CKD prevalence in the highest UHR quartile was 2.3 times that in the lowest quartile [24].

Turning to FPG, the highest quartile was associated with a 37% higher CKD risk than the lowest after adjusting for age and sex (*HR* = 1.37, 95%*CI*:1.06–1.77), but this association lost statistical significance after further covariate adjustment. This may be because the included covariates co-participate in metabolic disturbance-related renal injury, obscuring FPG’s independent effect—rather than negating its well-documented role as a CKD risk factor [25,26]. Notably, our RCS analysis uncovered a linear positive association between FPG and CKD risk (*P* for non-linear > 0.05). (Supplementary Figure S2). This linear trend suggests that even within the non-diabetic range, higher FPG levels are consistently linked to an increased risk of renal injury, echoing findings from previous population-based cohorts [27,28]. Thus, lower FPG levels within the normal range might be associated with better kidney health even in non-diabetic individuals, although interventional studies are needed to confirm this.

After establishing the independent effects of UHR and FPG, the rationale for employing GBMTM lies in its ability to transcend the limitations of single time-point or single-marker analyses by characterizing the dynamic, co-evolving patterns of UHR and FPG over time. This approach elucidates how their interaction over the long term jointly influences the risk of incident CKD. The dual-trajectory method aids in identifying population subgroups characterized by distinct risk evolution patterns, thereby providing a more comprehensive perspective on the complex relationship between metabolic disturbances and renal injury.

Based on this, the entire study population was classified into four trajectory groups: Group 1 (low UHR–low FPG), Group 2 (moderate UHR–moderate FPG), Group 3 (moderate–high UHR with fluctuating moderate FPG), and Group 4 (high UHR with fluctuating moderate FPG). The risk of CKD in Group 4 was 4.21 times that in Group 1 (*HR* = 4.21, 95% *CI*: 2.88–6.15). These results indicate that the dynamic interplay between UHR and FPG is substantially associated with an amplified risk of incident CKD. We further validated the aforementioned association through subgroup analyses. The results showed that across all subgroups except females, Group 4 consistently exhibited the highest risk of incident CKD, indicating that the joint trajectory pattern of high UHR and fluctuating fasting blood glucose is closely associated with incident CKD risk. Notably, a defining characteristic of Group 4 is high UHR levels, which correspond to markedly elevated SUA concentrations. Given that SUA levels in females are generally lower than those in males [29], the number of female participants in Group 4 was extremely small (*n* = 8, accounting for only 2% of the total sample in this group). This, coupled with the absence of incident CKD events within this female subgroup, meant that reliable hazard ratio estimates for female participants in Group 4 could not be obtained.

According to the CKD diagnostic criteria from the KDIGO guidelines, the incidence of CKD in the overall population included in this study was 7.40%, which is lower than that in a cohort study by Liu et al. exploring the correlation between CKD and UHR, where the CKD incidence was 13.13% [23]. A possible reason for this disparity is that the male proportion in our study population was 58.04%, while in the comparative study, it reached as high as 77%. Accumulating evidence has firmly established that men are independent risk factors for CKD, and they generally face a higher risk than women [30]. Men are more prone to be exposed to various CKD risk factors, including hypertension [31], obesity [32], and metabolic syndrome [33]. Moreover, testosterone may exacerbate kidney damage by enhancing the activity of the renin-angiotensin system (RAS) [34]. Consequently, disparities in the sex ratio might be one of the crucial factors contributing to the differences in CKD incidence. Additionally, variations in CKD diagnostic criteria could also play a significant role in these incidence discrepancies.

In this study, the GBMTM model was used to fit the dual trajectories of UHR and FPG, revealing their synergistic interaction pattern. Elevated UHR not only reflects excess SUA and compromised HDL-C antioxidant capacity, but may also exhibit bidirectional interplay with FPG fluctuations: high SUA levels exacerbate insulin resistance by inhibiting phosphorylation of Insulin Receptor Substrate 1 (IRS-1) [35], while oxidatively modified HDL-C exhibits impaired reverse cholesterol transport [36]; conversely, hyperglycemic environments activate the aldose reductase pathway promoting uric acid synthesis [37], suppress URAT1-mediated urate excretion, and induce glycosylation-modified HDL-C with diminished anti-inflammatory function [38,39]. In the dual-trajectory model, the high-UHR with moderate FPG fluctuation group (Group 4) exhibited a typical metabolic imbalance. The UHR level in Group 4 was 200% higher than that in Group 1, and the HDL-C level was only 0.91 ± 0.14 mmol/L. This imbalance may synergistically contribute to kidney injury through multiple potential pathways. Previous experimental models suggest that elevated SUA may activate the NLRP3 inflammasome, potentially triggering renal tubulointerstitial inflammation [40]. Furthermore, it is hypothesized that glycosylated HDL-C loses its ability to scavenge or sequester AGEs, thereby failing to attenuate the activation of the receptor for advanced glycation end-products (RAGE) [41]. This could lead to exacerbated RAGE-mediated oxidative stress [42]. The combination of these two factors may cause the reactive oxygen species (ROS) in renal tissue to exceed the clearance threshold [43], directly promoting apoptosis and fibrosis [44].

It is important to emphasize that the mechanistic interpretations discussed herein are primarily hypothesis-generating. As this is an observational study, our findings demonstrate epidemiological associations rather than causal relationships. While our joint trajectory analysis successfully identifies a high-risk metabolic phenotype, we cannot definitively conclude that trajectory-based monitoring or targeted interventions on UHR and FPG dynamics will directly prevent or delay the onset of CKD. Such clinical implications must be rigorously evaluated and confirmed in future prospective validation cohorts and interventional studies.

Limitations: (1) Residual confounding from unmeasured variables (e.g. dietary patterns, genetic polymorphisms) may persist.(2) Selection bias: Our health-checkup cohort may not fully represent the general population due to inherently higher socioeconomic status.(3) Lack of medication data could confound the observed biomarker associations.(4) Outcome misclassification: CKD detected at the final visit relied on a single measurement, potentially misclassifying transient kidney injury as CKD.

(5) Small sample sizes in specific subgroups (e.g. females in Group 4) limited statistical power for subgroup analyses. (6) Compromised temporality: A major limitation of this study is the overlapping temporal design. The trajectory groups were derived using biomarker data spanning the entire follow-up period, which included the period during which CKD events occurred. This design may introduce a potential bias of reverse causality. Although our stepwise sensitivity analyses excluding early incident cases demonstrated highly robust results, this design precludes strict causal sequencing. Therefore, our findings should be interpreted as robust long-term epidemiological associations rather than strict predictive causal pathways.

## Conclusions

In conclusion, this study constructed a dual-trajectory model of UHR and FPG. While the independent association of FPG with CKD risk was attenuated after full adjustment, our joint trajectory analysis demonstrated that the longitudinal pattern characterized by persistently high UHR accompanied by moderate FPG fluctuation was associated with the highest CKD risk. These findings are hypothesis-generating and suggest that combined dynamic assessment may help characterize high-risk metabolic phenotypes. However, in the absence of formal prediction-performance analyses or interventional data, the clinical utility of intensified monitoring or targeted preventive measures must be framed cautiously. Future research should seek to validate these trajectory phenotypes in prospective cohorts, and evaluate whether targeted interventions aimed at moderating these metabolic dynamics can effectively reduce CKD risk.

## Supplementary Material

Supplementary.docx

## Data Availability

The datasets generated and analyzed during the current study are derived from the internal health management database of Sichuan Provincial People’s Hospital. Due to ethical restrictions and institutional data management policies, the datasets cannot be made publicly available. Eligible researchers may request access to the data for legitimate research purposes by contacting the corresponding author:shuaiping@med.uestc.edu.cn.

## Declaration of generative Al and Al-assisted technologies in the writing process

During the preparation of this work the authors used ChatGPT in order to improve language. After using this tool, the authors reviewed and edited the content as needed and take full responsibility for the content of the publication.
