# Supplementary material for: Multi-trajectories of serum uric acid/high density lipoprotein cholesterol ratio and fasting plasma glucose in chronic kidney disease
Source: Ann Med. 2026 Jun 18;58(1):2689825. doi: 10.1080/07853890.2026.2689825 (PMC13288539; doi:10.1080/07853890.2026.2689825)
Supplement: Supplementary.docx [file IANN_A_2689825_SM6240.docx]

***Supplementary material***


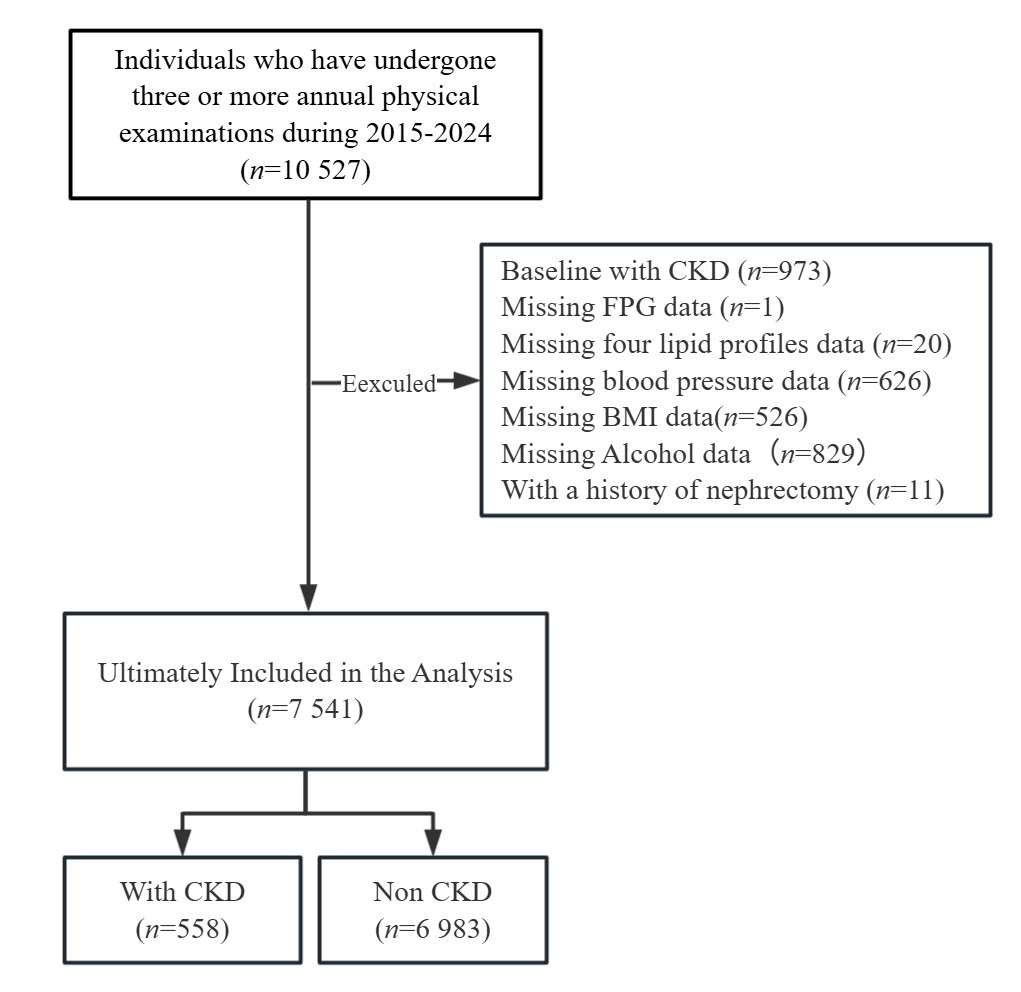


**Fig.S1** Flow diagram of participant selection

| A  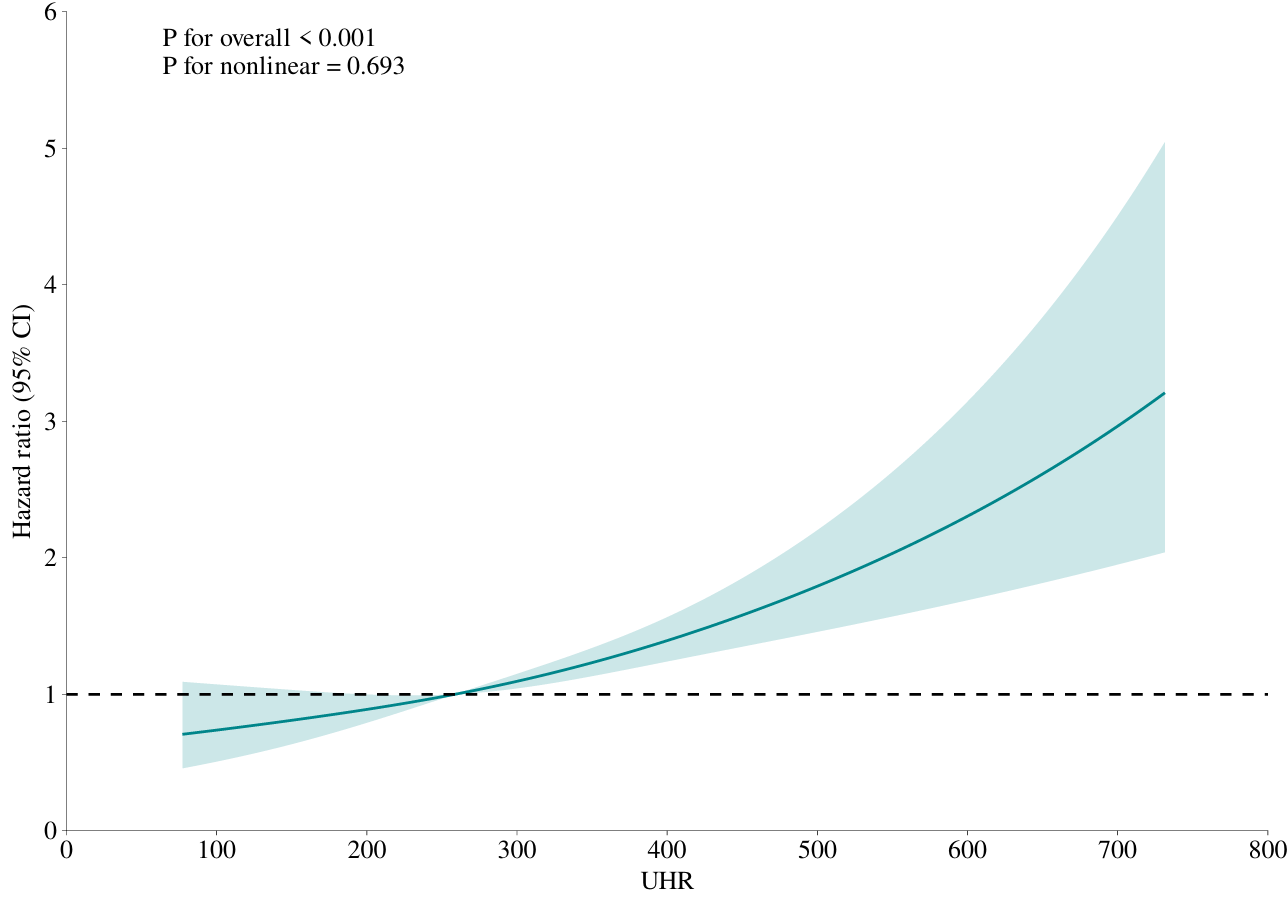 | B  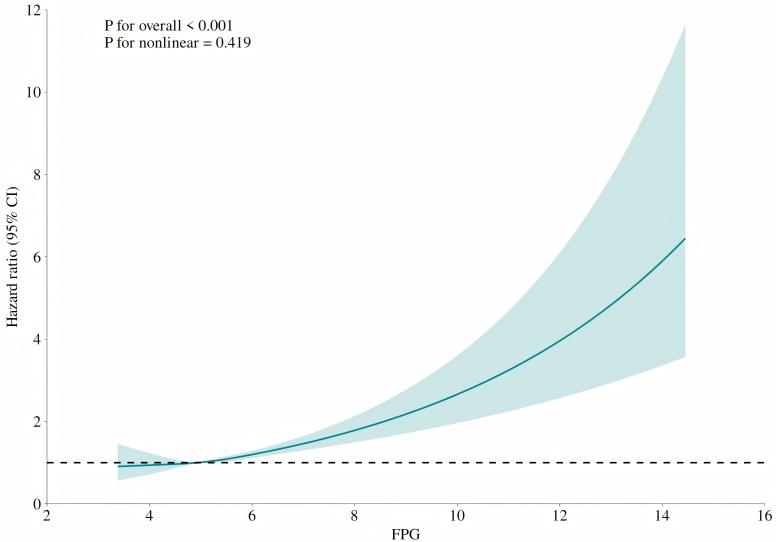 |
| --- | --- |

**Fig. S2** RCS analysis of UHR and FPG with the risk of developing CKD

Note: Adjust for age, sex, alcohol consumption, hypertension, SBP, LDL - C, TC, BMI, and BUN/Cr Ratio. When analyzing UHR, adjust for FPG; when analyzing FPG, adjust for UHR.The models used 3 knots(located at the 10th, 50th, and 90th percentiles), with reference values (*HR*=1) set at 258.73 for UHR and 4.93 mmol/L for FPG.

**Table S1**. Model selection criteria during the UHR-FPG Multi-trajectory model fitting process

| **Trajectory Groups** | **Degree of a polynomial** | ***BIC*** | ***AvepP*** | ***OCC*** | ***Class（%）*** |
| --- | --- | --- | --- | --- | --- |
| 1 | UHR(3）  FPG（3） | -203 380.6 |  |  | 100.0 |
| 2 | UHR（3，3）  FPG（3，3） | -195 821.3 | 0.972/0.953 | 18.17/38.22 | 65.4/34.6 |
| 3 | UHR（3，3，3）  FPG（3，3，3） | -192 831.9 | 0.960/0.928/0.945 | 25.42/22.16/98.97 | 48.2/36.87/14.9 |
| 4* | UHR（3，3，1，1）  FPG（0，0，3，3） | -191 613.2 | 0.945/0.895/0.914  /0.929 | 25.14/17.55/39.34  /233.95 | 40.7/32.8/21.2/5.3 |
| 5 | UHR（3，3，1，2，3）  FPG（3，3，1，0，3） | -186 140.8 | 0.959/0.914/0.958  /0.939/0.992 | 25.79/21.50/342.99  /109.46/11746.5 | 47.32/32.99/12.41/6.27/1.00 |

Note: * indicates the optimal model. BIC: Bayesian Information Criterion; AvepP: Average Posterior Probability; OCC: Odds of Correct Classification. In the SAS PROC TRAJ procedure, the BIC is calculated based on the log-likelihood and is output as a large negative value; a value closer to zero indicates a superior balance between model fit and parsimony. The modeling utilized the Censored Normal (CNORM) distribution. The polynomial order for each trajectory is defined by specific parameters (0 = intercept only, 1 = linear, 2 = quadratic, 3 = cubic). The optimal complexity for each trajectory was determined by evaluating the statistical significance (*P* < 0.05) of the parameter estimates, with non-significant higher-order terms systematically dropped. For the optimal 4-group model, the polynomial degrees were specified as (3, 3, 1, 1) for UHR and (0, 0, 3, 3) for FPG.

| **Table S2.** Trajectory Equation of the UHR-FPG Model | | |
| --- | --- | --- |
| **Variables** | ***Group*** | ***Equation*** |
| **UHR** | Group 1 | ŷ=176.173-6.220x+1.479x^2^-0.106x^3^ |
|  | Group 2 | ŷ=283.810-12.738x+2.703x^2^-0.199x^3^ |
|  | Group 3 | ŷ=390.481-5.481x+0.000x^2^-0.000x^3^ |
|  | Group 4 | ŷ=520.742-6.272x+0.000x^2^-0.000x^3^ |
| **FPG** | Group 1 | ŷ=4.889-0.000x+0.000x^2^-0.000x^3^ |
|  | Group 2 | ŷ=5.354-0.000x+0.000x2-0.000x3 |
|  | Group 3 | ŷ=5.372-0.036x+0.041x^2^-0.004x^3^ |
|  | Group 4 | ŷ=5.286-0.077x+0.066x^2^-0.007x^3^ |

**Table S3.** Trajectory Parameter Estimation of the Model

| **Variables** | ***Parameter*** | ***Group*** | ***β*** | ***SE*** | ***t*** | ***P*** |
| --- | --- | --- | --- | --- | --- | --- |
| **UHR** | Intercept | Group 1 | 176.17328 | 1.15467 | 152.574 | 0.0000 |
|  | Linear | Group 1 | -6.22031 | 1.50355 | -4.137 | 0.0000 |
|  | Quadratic | Group 1 | 1.47911 | 0.55523 | 2.664 | 0.0077 |
|  | Cubic | Group 1 | -0.10569 | 0.05248 | -2.014 | 0.0440 |
|  | Intercept | Group 2 | 283.80964 | 1.85455 | 153.034 | 0.0000 |
|  | Linear | Group 2 | -12.79807 | 1.67125 | -7.622 | 0.0000 |
|  | Quadratic | Group 2 | 2.70293 | 0.58037 | 4.578 | 0.0000 |
|  | Cubic | Group 2 | -0.19930 | 0.05470 | -3.644 | 0.0003 |
|  | Intercept | Group 3 | 390.48060 | 2.10489 | 185.511 | 0.0000 |
|  | Linear | Group 3 | -5.48064 | 0.33245 | -16.486 | 0.0000 |
|  | Intercept | Group 4 | 520.74173 | 3.23305 | 161.069 | 0.0000 |
|  | Linear | Group 4 | -6.27215 | 0.65941 | -9.512 | 0.0000 |
| **FPG** | Intercept | Group 1 | 4.88851 | 0.01235 | 395.829 | 0.0000 |
|  | Intercept | Group 2 | 5.35387 | 0.01689 | 317.004 | 0.0000 |
|  | Intercept | Group 3 | 5.37182 | 0.03307 | 162.448 | 0.0000 |
|  | Linear | Group 3 | -0.03607 | 0.04102 | -0.879 | 0.3793 |
|  | Quadratic | Group 3 | 0.04103 | 0.01399 | 2.933 | 0.0034 |
|  | Cubic | Group 3 | -0.00441 | 0.00124 | -3.562 | 0.0004 |
|  | Intercept | Group 4 | 5.28624 | 0.05918 | 89.321 | 0.0000 |
|  | Linear | Group 4 | -0.07878 | 0.08205 | -0.968 | 0.3494 |
|  | Quadratic | Group 4 | 0.06587 | 0.02826 | 2.331 | 0.0197 |
|  | Cubic | Group 4 | -0.00662 | 0.00248 | -2.668 | 0.0076 |

**Table S4.** Baseline characteristics of study participants by UHR-FPG Multi-trajectory groups

| Characteristics | Group 1  (n = 3072) | Group 2  (n = 2473) | Group 3  (n = 1598) | Group 4  (n = 398) | Statistic | *P* |
| --- | --- | --- | --- | --- | --- | --- |
|  |  |  |  |  |  |  |
| Age, years | 45.88 ± 14.38 | 53.14 ± 13.90 | 52.60 ± 12.94 | 50.21 ± 13.41 | F=151.00 | **<0.001** |
| SBP, mmHg | 115.58 ± 15.10 | 123.06 ± 16.11 | 124.42 ± 15.75 | 124.10 ± 15.46 | F=164.26 | **<0.001** |
| DBP, mmHg | 68.89 ± 9.71 | 73.36 ± 10.41 | 75.85 ± 10.49 | 76.29 ± 11.36 | F=208.31 | **<0.001** |
| BMI, kg/m^2^ | 21.92 ± 2.60 | 24.20 ± 2.67 | 25.51 ± 2.71 | 26.33 ± 2.90 | F=859.89 | **<0.001** |
| TC, mmol/L | 4.75 ± 0.91 | 4.82 ± 0.91 | 4.80 ± 0.91 | 4.63 ± 0.89 | F=6.19 | **<0.001** |
| TG, mmol/L | 1.09 ± 0.56 | 1.62 ± 1.07 | 2.21 ± 1.58 | 2.85 ± 2.04 | F=540.11 | **<0.001** |
| LDL-C, mmol/L | 2.63 ± 0.77 | 2.90 ± 0.80 | 2.91 ± 0.79 | 2.69 ± 0.79 | F=75.53 | **<0.001** |
| HDL-C,mmol/L | 1.59 ± 0.28 | 1.27 ± 0.19 | 1.07 ± 0.17 | 0.91 ± 0.14 | F=2558.00 | **<0.001** |
| SUA, μmol/L | 273.15 ± 50.61 | 357.02 ± 57.10 | 418.03 ± 59.40 | 475.97 ± 70.24 | F=3365.21 | **<0.001** |
| UACR, mg/g | 8.40 ± 5.68 | 7.69 ± 5.42 | 7.60 ± 5.52 | 7.91 ± 5.39 | F=10.65 | **<0.001** |
| eGFR, ml/min | 108.11 ± 15.33 | 98.25 ± 14.56 | 96.22 ± 14.44 | 95.72 ± 15.27 | F=327.42 | **<0.001** |
| BUN，mmol/L | 4.97 ± 1.24 | 5.28 ± 1.25 | 5.39 ± 1.23 | 5.30 ± 1.27 | F=50.41 | **<0.001** |
| SCr, umol/L | 59.12 ± 12.15 | 72.21 ± 13.54 | 77.68 ± 11.92 | 80.87 ± 11.68 | F=1058.26 | **<0.001** |
| UHR | 176.40 ± 40.74 | 285.87 ± 49.62 | 398.02 ± 64.18 | 530.54 ± 90.50 | F=9596.60 | **<0.001** |
| FPG, mmol/L | 4.83 ± 0.57 | 5.28 ± 1.26 | 5.38 ± 1.35 | 5.26 ± 1.07 | F=134.62 | **<0.001** |
| Male, n (%) | 639 (20.80) | 1851 (74.85) | 1495 (93.55) | 392 (98.49) | χ²=3131.26 | **<0.001** |
| Hypertension, n (%) | 320 (10.42) | 662 (26.77) | 465 (29.10) | 109 (27.39) | χ²=333.67 | **<0.001** |
| Type 2 diabetes, n (%) | 84 (2.73) | 271 (10.96) | 210 (13.14) | 32 (8.04) | χ²=204.40 | **<0.001** |
| Hyperuricemia, n (%) | 66 (2.15) | 453 (18.30) | 776 (48.44) | 315 (79.55) | χ²=2185.93 | **<0.001** |
| Dyslipidemia, n (%) | 1096 (35.68) | 1393 (56.33) | 1310 (81.98) | 383 (96.23) | χ²=1210.17 | **<0.001** |
| Alcohol, n (%) | 185 (6.02) | 422 (17.06) | 378 (23.65) | 96 (24.12) | χ²=331.93 | **<0.001** |

**Table S5.** Sensitivity analyses for the association between trajectory groups and incident CKD risk

| **Trajectory Groups** | **Model 1**  ***HR* (95% *CI*)** | ***P*** | **Model 2**  ***HR* (95% *CI*)** | ***P*** |
| --- | --- | --- | --- | --- |
| Group 1 | 1.00 (Reference) |  | 1.00 (Reference) |  |
| Group 2 | 1.19 (0.92 ~ 1.54) | 0.190 | 1.18 (0.72~1.93) | 0.510 |
| Group 3 | 1.70 (1.24 ~ 2.33) | **<0.001** | 2.19 (1.26 ~3.79) | **0.005** |
| Group 4 | 3.33 (2.24 ~ 4.96) | **<0.001** | 4.95 (2.49 ~ 9.83) | **<0.001** |

Note: To robustly address the potential risk of reverse causality due to the overlapping temporal design, stepwise sensitivity analyses were performed. Model 1 excluded early incident cases diagnosed at the second follow-up visit, as these participants lacked the minimum longitudinal data points required to robustly define a pre-disease trajectory. Model 2 further restricted the cohort by additionally excluding incident cases diagnosed at the third follow-up visit to ensure a stricter temporal sequence. Both models were fully adjusted for age, sex, alcohol consumption, hypertension, SBP, LDL-C, TC, BMI, and BUN/Cr Ratio.
